# Fish learn collectively, but groups with differing personalities are slower to decide and more likely to split

**DOI:** 10.1242/bio.033613

**Published:** 2018-05-01

**Authors:** Kyriacos Kareklas, Robert W. Elwood, Richard A. Holland

**Affiliations:** 1 School of Biological Sciences, Medical Biology Centre, Queen's University Belfast, 97 Lisburn Road, Belfast, Northern Ireland, BT9 7BL, UK; 2 School of Biological Sciences, Bangor University, Deiniol Road, Bangor, Gwynedd, LL57 2UW, UK

**Keywords:** Collective cognition, Decision-making, Personality, Spatial learning, Shoaling

## Abstract

We tested zebrafish shoals to examine whether groups exhibit collective spatial learning and whether this relates to the personality of group members. To do this we trained shoals to associate a collective spatial decision with a reward and tested whether shoals could reorient to the learned location from a new starting point. There were strong indications of collective learning and collective reorienting, most likely by memorising distal cues, but these processes were unrelated to personality differences within shoals. However, there was evidence that group decisions require agreement between differing personalities. Notably, shoals with more boldness variation were more likely to split during training trials and took longer to reach a collective decision. Thus cognitive tasks, such as learning and cue memorisation, may be exhibited collectively, but the ability to reach collective decisions is affected by the personality composition of the group. A likely outcome of the splitting of groups with very disparate personalities is the formation of groups with members more similar in their personality.

## Abstract (precis)

**Summary:** Zebrafish groups were trained to make collective decisions and memorised locations by using cues. However, collective decisions were made slower by the splitting of groups comprising individuals of dissimilar personality.

## INTRODUCTION

Organised groups are characterised by cooperative and synchronised behaviour, which allows for better resource acquisition and risk avoidance (Pitcher and Parrish, 1993). However, collective behaviour varies depending on external and internal conditions, e.g. environmental risk levels and inter-group dynamics (Hoare et al., 2004; Sumpter, 2006). On some occasions, such as during foraging, this may require that information about current local conditions is disseminated between individuals within the group and presumably processed collectively by the group (Laland and Williams, 1997). The collaborative use of shared information to solve problems and make decisions is called collective cognition (Couzin, 2009). Although collective cognition may be utilised for various group functions, it is particularly useful for adjusting group behaviour in spatial contexts such as food location or route choice (de Perera and Guilford, 1999; Conradt and Roper, 2005; Couzin et al., 2005). Indeed, group living has been proposed to enhance navigation performance via information-sharing (Simons, 2004). Navigation relies on several behavioural and cognitive processes, such as exploration/sampling effort, decision-making, learning and cue memorisation (Brown et al., 2006). The use of these processes by a group may be limited by the extent to which cognitive or behavioural similarities between individuals facilitate collective responses.

Most studies on group navigation have focused on collective decision-making as a means of choosing between routes while maintaining group structure (Couzin, 2009; Couzin et al., 2005; Conradt and Roper, 2005). Yet individual variation has been noted in important cognitive processes: some individuals may be better at memorising information from their environment (Croston et al., 2016), faster or more successful in their decisions (Chittka et al., 2009) or faster learners (Trompf and Brown, 2014). Interestingly, individual variation in many of these processes has been linked to animal personality (Griffin et al., 2015; Guillette et al., 2016). Animal personality is often described by behavioural traits exhibiting consistent inter-individual differences and intra-individual repeatability (Wolf and Weissing, 2012). A well-studied trait, boldness, is indicated by exploration tendencies and feeding motivation (Toms et al., 2010), making it a regular predictor of spatial associative learning (e.g. Trompf and Brown, 2014; Mamuneas et al., 2015). Although a prominent hypothesis is that bolder animals are faster but less accurate in their decisions (Chittka et al., 2009), often effects manifest independently of these trade-offs. For example, bolder fish may be faster at choosing between locations and faster learning rewarded responses, but not less accurate in their choices than more timid animals (Trompf and Brown, 2014; Mamuneas et al., 2015; Kareklas et al., 2017). Regardless of these trade-offs, the effects of personality on cognitive performance may also influence how animals work collectively. In particular, personality differences between individuals may predict how they tackle cognitive tasks collectively; the exploration tendencies and reward motivation of group members could affect how they coordinate responses, how they decide, and how they organise, share and utilise information when learning (Couzin, 2009).

**Fig. 1.**
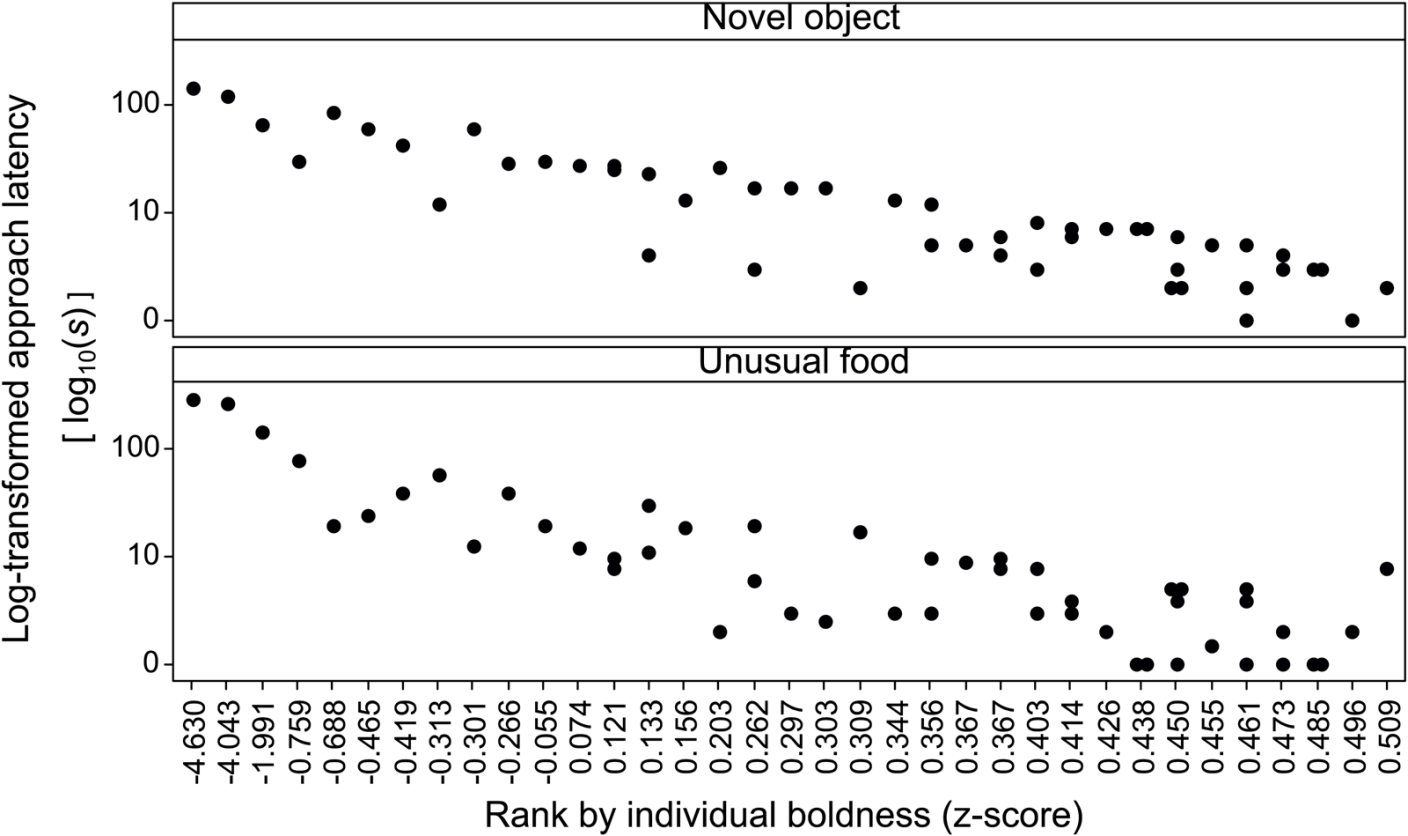
**Latency distributions on a logarithmic scale for the novel-object and feeding test, as exhibited by individuals (*n*=50) ranked by their composite boldness score.**

To examine whether collective processes of decision-making and learning are affected by the composition of groups, in terms of the individual boldness of their members, we studied the zebrafish *Danio rerio*. Fish were first tested as individuals to determine their levels of boldness (Fig. 1) and were then trained as groups of five, referred to here as shoals, in a spatial-associative learning task. During training, only spatial decisions made by all individuals by reaching a location together were reinforced (reward or punishment), to determine learning specific to a collective response. After reaching a learning criterion, we tested the ability of shoals to reorient, examining their ability to memorise distal cues during training. Animals may simply rely on the memorisation of a response, such as a turning direction, or also on the memorisation of the relative positions of distal cues (Tolman et al., 1946; Burgess, 2006). Associating a memorised response to a rewarded location relies on orienting from a familiar starting point. In contrast, the additional memorisation of distal cues can facilitate reorientation from novel starting points by attending to changes in the relative position of these cues towards the correct location (place learning; Rodriguez et al., 1994). Therefore, reorienting can identify whether learning relies on composite strategies that utilise the memorisation of the relative position of distal cues or simple associations of location to directional-response.

First, we tested the hypothesis that collective decisions, learning and memorisation are related to mean boldness levels, with shoals of bolder composition differing from those with shier composition. Second, we tested the hypothesis that collective decisions, learning and memorisation are predicted by the variance in boldness among shoal members, because large differences in personality inhibit agreement or cooperation. Based on effects by personality composition on group response time in other shoaling species, we expected decision times to be related to boldness, being generally faster for groups of bolder individuals (Dyer et al., 2009). The learning of a collective response and memorisation strategies, such as place learning, have only recently been experimentally studied in fish groups (McAroe et al., 2017), noting both the facilitation of visual-cue memorisation and faster learning by zebrafish in groups. However, the effects of the personality composition of groups on these group processes have not been examined. We predict that links to personality may be indicated due to either differences between individuals in their response tendency or their performance in particular cognitive tasks, with more variable groups reaching lower agreement and cohesion (Ioannou and Dall, 2016), and overall bolder groups being faster to decide and associate food reward to a location [such as in individuals, e.g. Griffin et al. (2015); Guillette et al. (2016); Kareklas et al. (2017)].

## RESULTS

### Collective decisions

**Fig. 2.**
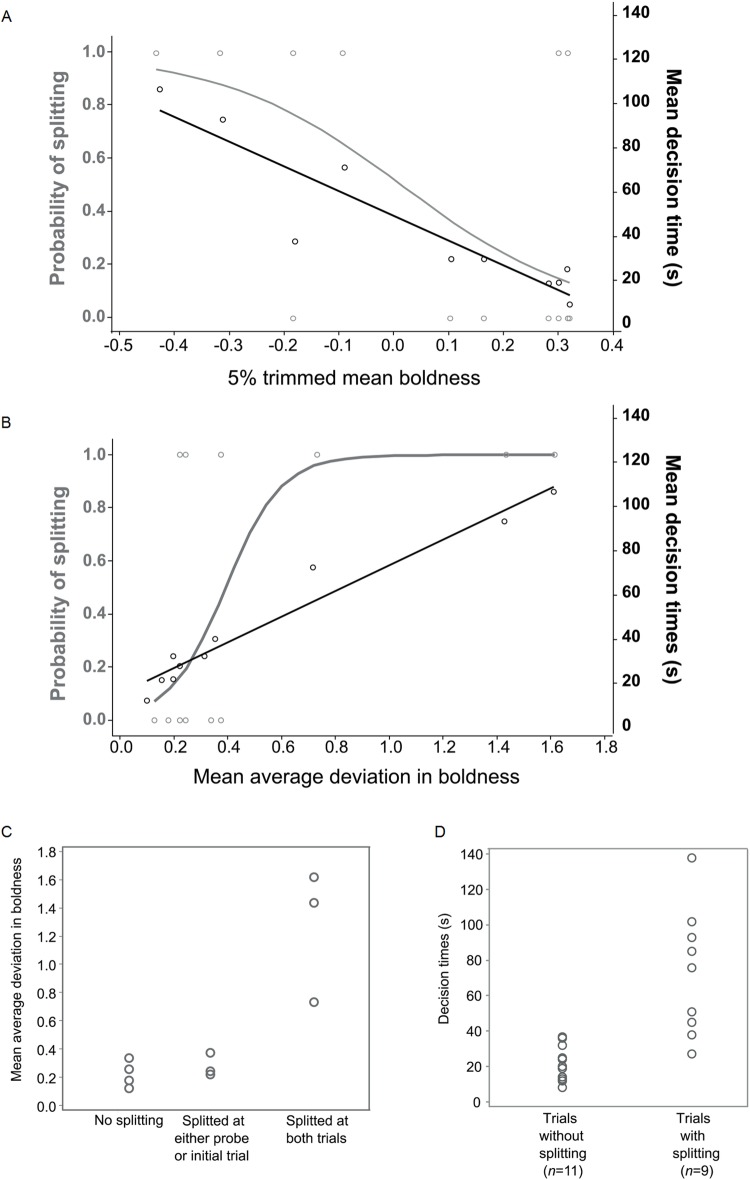
**Shoal cohesion (probability of splitting) and consequent effects on collective decision-times were influenced by individual boldness differences, but were not linked to majority averages in boldness.** (A) The mean boldness of shoal members (5% trimmed to exclude biases by extremely bold or timid fish) had a negative, non-significant, effect on mean decision times between initial and probe trial (black line and marks), but no effect on splitting probability (grey curve and marks) as indicated by regression models (decision times: linear, probability of splitting: binomial). (B) In contrast, the variance in boldness within shoals (mean average deviation of all fish) positively predicted the probability of splitting at probe and initial trials (grey curve and marks) and the mean decision times between initial and probe trial (black line and marks). (C) The level of consistency in splitting between initial and probe trials was greater for shoals with higher variance in boldness (Zero splitting:mean MAD=0.225, one trial: mean MAD=0.279, two trials: mean MAD=1.26; ANOVA, *P*<0.01) and (D) shoals took longer to reach a decision if they split (split: mean=21.82±3 s.e.m., no split: mean=72.8±12 s.e.m.; Welch's *t*, *P*<0.01).

All shoals reached collective decisions within the time limit (<5 min) in both the initial and probe trial, but some tended to split before reaching a decision (please see the supplemental information). No significant differences were found between the initial trial (before training) and the probe trial (after training) for either decision times (*R^2^*=0.017; *P*>0.05) or the probability of splitting (*R^2^*=0.02; *P*>0.05), suggesting consistency in collective behaviour and limited effects from differing individual learning during training. The mean boldness of shoal members did not significantly contribute to the probability of splitting (*R^2^*=0.016; *P*>0.05; Fig. 2A), and although shoals with members of greater mean boldness exhibited shorter decision times (*R^2^*=-0.73; Fig. 2A) the relative effect was not significant (*P*>0.05). The only significant predictor was variance in shoal-member boldness, which strongly predicted both collective decision-times (*R^2^*=0.816; *F_1,20_*=9.19, *P*=0.008) and the probability of splitting (*R^2^*=0.482, χ*^2^_1,20_*=13.26, *P*<0.001). Groups with greater variance in boldness between their members were more likely to split and took longer to collectively reach an arm (Fig. 2B). Further, consistency in splitting across trials was noted for shoals with greater variance in boldness (ANOVA, *F_3,10_*=15.93, *P*=0.002, *R^2^*=0.820; Fig. 2C) and collective decisions took longer when splitting occurred than when not (Welch's *t*=4.15, *P*=0.002; Fig. 2D).

Decision accuracy (number of erroneous decisions during training) was only weakly predicted by the mean of shoal-member boldness (*R^2^*=0.127; *χ^2^*=8.19, *P*<0.05), but was not significantly predicted by the probability of splitting decision (*R^2^*<0.04; *P*>0.05). Contrary to predicted speed–accuracy trade-offs (Chittka et al., 2009), the number of erroneous decisions during training did not significantly correlate with the time shoals needed to decide in either the initial or the probe trial (*r_s_*<0.2, *P*>0.05).

### Collective learning

**Fig. 3.**
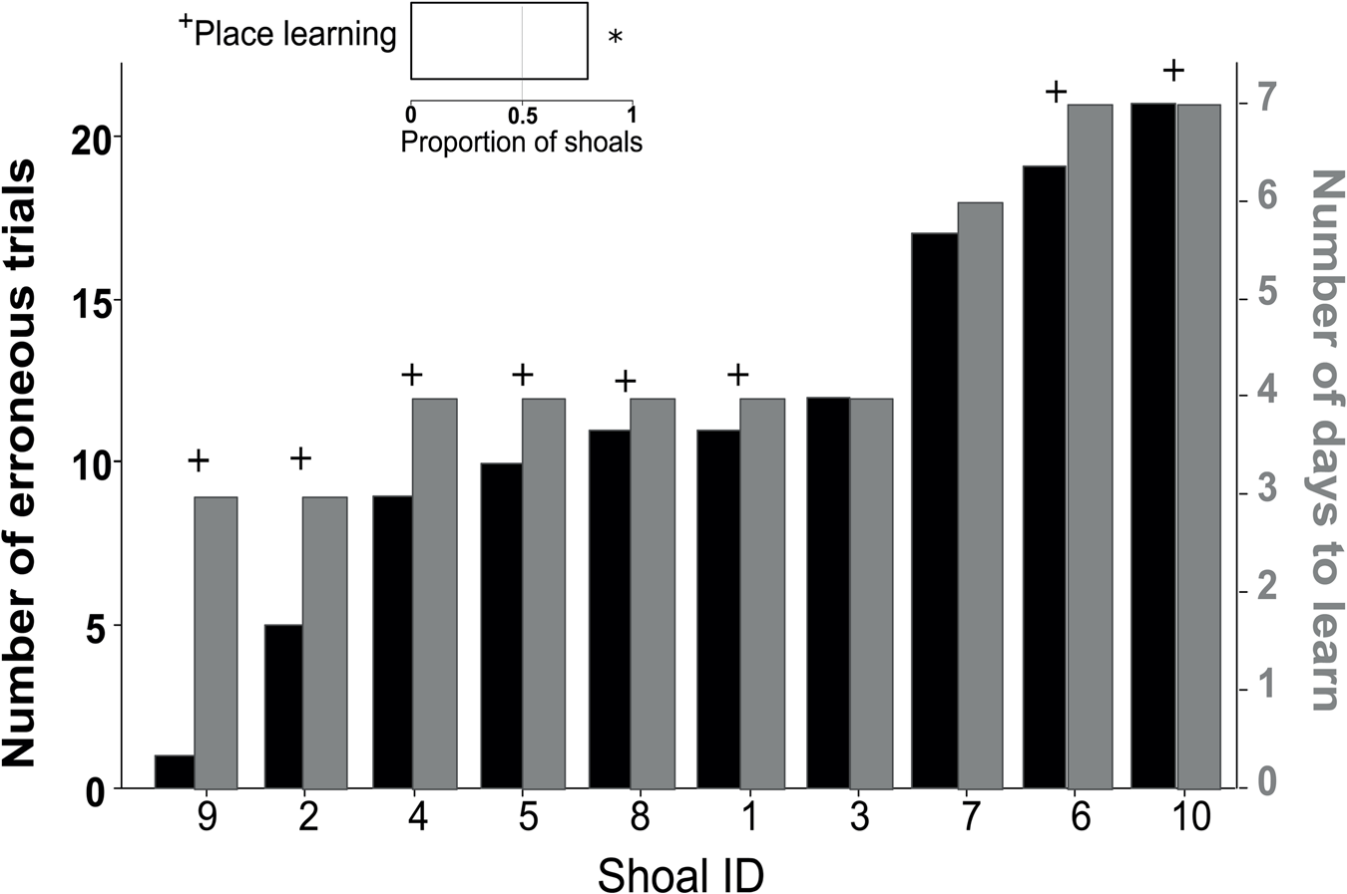
**Shoals that made more erroneous trials during training (black bars) also took more days to learn (grey bars), but a greater than chance majority of shoals was able to memorise place.** Inset: proportion of shoals reorienting at probe trial, showing place learning. Shoals (*n*=10) are ordered by increasing number of error counts and marked (cross) if they showed place learning (*P<0.05, binomial-test).

All shoals met the collective learning criterion of all fish being simultaneously in the rewarded location for eight/ten trials over three consecutive days (Fig. 3). The rate of learning (number of days to reach criterion) was negatively related to the number of erroneous choices during training (i.e. choosing the punished arm) (*R^2^*=−0.945, *χ^2^_1,10_*=3.99, *P*=0.046; Fig. 3). However, learning rate was not significantly predicted by the variance and the mean of shoal-member boldness, or the likelihood of splitting (*R^2^*<0.04; *P*>0.05).

At probe trials from the new starting point in the top arm, which was blocked during training, all shoals reached one of the arms collectively (i.e. were at the same arm together before the 5 min), but the ability to reorient to the arm rewarded during training was unrelated to the variance and the mean of shoal-member boldness or the likelihood of splitting (*R^2^*<0.04; *P*>0.05). Indeed, the majority of shoals (eight/ten) showed preference for reaching the rewarded arm significantly more than predicted by chance (proportion>0.5: *z_10_*=1.90, *P*=0.029; Fig. 3).

## DISCUSSION

To collectively reach one of two locations, groups must maintain cohesion and structure. This relies on interactions between the individuals comprising the group, a process known as self-organisation (Sumpter, 2006). The interactions facilitate information sharing (Couzin, 2009; Ward et al., 2011) and in fish this can be in the form of changes in swimming direction, where swimming towards a location by some individuals propagates through the group (Croft et al., 2003). The extent of the propagation is indicated by the time needed by all individuals to change direction together, which can be limited by individuals deciding to act otherwise (Couzin, 2009; Ward et al., 2008). Here, our findings implicate personality differences between group members in this process. Groups with greater variance in boldness between their members were consistently more likely to split and took longer to collectively reach an arm (Fig. 2B,C). Given collective decisions took longer when splitting occurred than when not (Fig. 2D), we conclude that the splitting of groups with members more dissimilar in their boldness results in collective decisions taking longer to be reached. The involvement of personality on collective decision speed may reflect a greater tendency by bolder individuals to reach food-rewarded locations (Kareklas et al., 2017).

The relationship of personality differences with cohesion and collective-decision speed proposes that high-variance groups might be disadvantaged when competing for spatially distributed resources. A study on guppies *Poecilia reticulata* did not find mixed groups more disadvantaged than bold groups, but faster at reaching food than shy groups (Dyer et al., 2009). Differences in the effects of personality may depend on the species, but the study in guppies also utilised a categorical separation of bold and shy to compose groups. In contrast, here we measured the variance in boldness score within randomly assembled groups. A higher variance in our shoals is most likely due to the presence of extremely shy individuals, according to individual latency distributions (Fig. 1). The direct effects of high variance on splitting are unclear, as we did not track individuals, but they are possibly driven by intra-group differences in exploration and approach tendency between more greatly differing personalities (Toms et al., 2010) and possibly due to related differences in sociality (Ward et al., 2004; McDonald et al., 2016). Another possibility is that differences in boldness correspond to differences in decision-making strategy (Griffin et al., 2015; Kareklas et al., 2017), which again would require identifying consistencies in the position individuals occupy in a shoal. Further, different types of splitting may represent different processes. Lateral fission may reflect individuals being less social and actively seeking to split, but rear fission may be the result of either active splitting or passive restraints (Croft et al., 2003), such as being more fearful and timid (Toms et al., 2010; Kareklas et al., 2017). The splitting of groups with very high variance in personality could possibly lead to the formation of groups with lower variance in personality. While this is yet to be tested, it could be a way for groups to ensure that agreements are reached more easily. Indeed, larger differences in personality can manifest effects on the way fish socialise, cooperate and prioritise reward or risk (Ioannou et al., 2015). Alternatively, splitting might be an effect of hierarchical dynamics, with leader initiations and follower delays relying on similarities in personality aspects such as boldness and flexibility (Ioannou and Dall, 2016).

Contrary to expectations that personality differences have an effect on both speed and accuracy due to trade-offs (Chittka et al., 2009), the number of erroneous decisions during training was independent of how fast fish in a shoal reached a location together. However, shoals that made fewer erroneous collective decisions during training reached the learning criterion faster (Fig. 3). This negative association between erroneous trials and learning rate is consistent with learning by positive reinforcement, given less erroneous shoals would collectively reach the rewarded arm more frequently during training (Brown et al., 2006), but suggests a low effect from negative reinforcement by the mild punishment of erroneous trials. Interestingly, the majority of shoals (eight/ten) re-oriented at probe trials to the location rewarded during training (Fig. 3). This indicates that most shoals did not simply use a learned response for collectively reaching the rewarded arm, e.g. turn direction, but learned the place of the reward. Place learning proposedly involves allocentric processes, where positions of distant cues in relation to a target are memorised and reorientation is possible (Tolman et al., 1946; Rodriguez et al., 1996). Although this may involve cognitive mapping (mental representations of space using the relative positions of landmarks), other cue-based strategies are difficult to exclude, e.g. beaconing to large cues near the goal (Bennett, 1996). Most notably, *D. rerio* zebrafish individuals can take longer to learn and do not prefer place over response learning (McAroe et al., 2016). Thus, being in a shoal can facilitate both learning efficiency and the use of learning strategies that rely on the memorisation of cues and not solely of simple directional responses. This has been exemplified recently in a study comparing shoals to individual zebrafish, where only shoals were able to exhibit place learning (McAroe et al., 2017). This is enabled in fish groups by social learning (Laland and Williams, 1997; Trompf and Brown, 2014), cooperative vigilance and information sharing (Pitcher and Parrish, 1993; Miller and Gerlai, 2011).

In contrast to models predicting that cohesion and individual differences in behaviour may affect collective behaviour and learning (Couzin, 2009), we found no strong evidence of personality or splitting having any significant influence on collective learning or accuracy. Decision accuracy and learning may instead be influenced by inter-individual differences in experience, attention, acquisition and cue perception (Couzin, 2009; Kao et al., 2014). Indeed, in the absence of effects from individual behavioural phenotypes, based on personality, differences in individual experience and a balancing between personal and shared information in the group are both very likely alternative factors (Miller et al., 2013). Otherwise, groups may rely on the leadership of more experienced or reward-driven individuals (de Perera and Guilford, 1999; Krause et al., 2000). For memorisation strategies in particular, there is evidence that individuals can use cue and response based strategies together and often animals reverse between strategies over training times (Packard and McGaugh, 1996; Burgess, 2006). These processes could carry over in collective learning and this can be tested by repeated probe trials during collective training.

Although our study did not include analysis of any kinematic data, recent work has increasingly shown the benefit of identifying behaviour-specific movement bouts (Marques et al., 2018) and for assessing how the solitary movement patterns of group members affect collective swimming patterns (Marras et al., 2015). This would provide more evidence for the individual effects on collective decisions and learning, and could identify the extent to which effects from individual motor behaviour are related to personality [e.g. bouts related to risk response or approach; Marques et al. (2018)] or other phenotypic factors, such as morphology (Conradsen and McGuigan, 2015). While these effects remain to be examined, here we show that zebrafish can learn to reach collective spatial decisions for rewards and utilise place memorisation strategies to do this, but that collective decisions are biased by personality differences.

## MATERIALS AND METHODS

### Animals and housing

Naïve adult male zebrafish *D. rerio* (*n*=50) were acquired from a local supplier, Grosvenor Tropicals, Lisburn, Northern Ireland. Given the supplier was not informed on strain variations in their stock, we used only males that show no strain preferences for shoaling (Snekser et al., 2010), which also removed the chance of mating during group living and controlled for sex-related differences in boldness. Fish were housed in tanks (26 cm W×36 cm L×30 cm H; 26±2°C and 7.4±0.4 pH dechlorinated tap water) enriched with fine sediment, plants and plastic pipes. Photoperiods were 12 h long (0700–1900) and feeding was daily (TetraMin® flakes).

### Behavioural tests for boldness

Following a week-long acclimation to individual housing (tanks filled to 15 L with view of neighbours to reduce isolation effects), the boldness of each fish was assessed in their housing tank by measuring consistency in their approach latency towards novelty between two contexts often used to test differences in boldness [see review by Toms et al. (2010)]. First, novel-object inspection was tested by the time fish took to reach ∼1.5 body-length distance from a 7 cm toy after it was lowered by a pulley system to the bottom of the tank, as estimated by viewing through a screen with a grid from above. Second, feeding motivation towards an unusual food was tested by recording the time fish needed to initiate feeding on chironomid larvae (released by forceps), which had not been previously offered to the fish in the laboratory. Opaque sheets visually separated each group from the others and shielded the observer during tests. Observations were made via a Sony HDR CX190E handycam video camera. Fish had not been fed for ∼24 h prior to testing. Both tests were 5 min in duration, carried out at 11:00–13.00, with a 48 h interval between them and in the same order for all fish to control for carry-over effects [see Kareklas et al. (2017)]. As would be expected for the expression of personality traits, like boldness (Toms et al., 2010; Wolf and Weissing, 2012), latencies were found to be consistent between contexts (Chronbach's *α*=0.803; Pearson's *r*=0.844) and used to calculate composite boldness scores. Greater latencies are linked to lower boldness (Toms et al., 2010), thus the standardised sums of latencies from both tests were used as scores (z-values) and inversed in sign (positive or negative) to rank by increasing boldness (Fig. 1).

### Collective tests for learning

Following individual behavioural tests, fish were randomly sorted in shoals of five (*n*=10) and housed together (tanks filled to 25 L) for a further week and then trained in a plus maze (four-arm maze constructed from acrylic sheets; each arm measuring 15 cm W×30 cm L). During training internal landmarks were unavailable, but visual cues were available outside the maze, including white paper sheets on a distant wall, adjacent tank tops and the camera arm above the tank. To control for inter-shoal differences by differing information, these external cues and their locations were kept constant during trials and for all shoals. Shoal trials started in the bottom arm and the top arm was blocked during training. Trials commenced by removing an opaque divider that kept shoals constrained in the starting arm for 2 min. Shoals were then presented with the two remaining arms, left or right, with 5 min to choose between them. A collective decision was indicated by all individuals being in the same arm at the same time, training them to associate a collective decision towards one arm with a reward and towards the other arm with a mild punishment. The choice of direction, left or right arm, for the rewarded and mildly-punished arm was randomised across shoals. When reaching the arm randomly assigned to be food rewarded, shoals were blocked in until each fish received 1–2 chironomid larvae (individual feeding latency was <5 s). However, in the unrewarded arm they were blocked in for 2 min and not fed [mild punishment; McAroe et al. (2016); Kareklas et al. (2017)]. Following their choice, fish were gently guided by a net to the starting arm. After each trial, the tank water was disturbed to minimise use of olfactory cues. Shoals had ten such trials daily until reaching a learning criterion of a minimum of eight/ten correct trials (i.e. collectively choosing the rewarded arm) on three consecutive days. The learning criterion corresponds to a learning plateaux and success rates exceeding 24/30 correct trials, which differ from chance (15/30) at the 0.1% level. Shoals were given a single probe trial 24 h after reaching the learning criterion, which started from the previously blocked top arm. This tested if fish were able to collectively reorient to the rewarded arm from a novel starting point, via the memorisation of the relative positions of the distal cues during training (Rodriguez et al., 1994). The probe trial was unrewarded to control for the use of olfactory cues.

Reaching the correct arm during probe trials showed the ability to reorient by using distal landmarks, i.e. place learning. By contrast, a failure to reach the goal arm in the probe trial was considered the result of learning to go left or right during training, i.e. response learning (McAroe et al., 2016, 2017). Collective decision speed, measured until the last fish of the group passed the mark to either arm (given all other fish were already in the same arm to designate a collective choice), was recorded only for the first training trial (novel task) and the probe trial (novel starting point). The choice of using decision times only from these two trials was because their novelty controlled possible effects of familiarity and experience of making a particular decision; decisions from other trials during training could be biased by reinforcement from previous trials and thus not representative of a novel decision. In addition, by measuring times at two relatively novel trials, where one was before and one after training, allowed us to examine if novel decisions are affected by the experience of training as a group. Comparisons before and after training further enabled us to test consistency in the effects of intra-group boldness on decision-making and to test for effects by individual-level learning. Before reaching collective decisions in these trials, some shoals exhibited splitting: individuals either stayed behind in the starting arm while others had chosen between left or right (rear fission) or went in a different direction, reaching the opposite arm from the rest (lateral fission) (Croft et al., 2003). The distance needed to travel between arms (centre to centre) was ∼27 cm or five zebrafish body-lengths (4–6 cm), and was thus considered sufficient to indicate splitting. We recorded the occurrence of any type of splitting as an inverse measure of cohesion. If fish reached an arm together within the 5 min recording time, any splitting was noted and the collective decision was recorded as either correct (rewarded arm) or erroneous (unrewarded arm). Alternatively, if no choice was reached, any splitting was again recorded, but we did not count the trial as either correct or erroneous. Decision accuracy was measured by the total number of erroneous trials throughout training, because the number of correct trials can also be influenced by fish not choosing. The number of training days to reach criterion indicated learning rate.

### Analysis

Calculations, analyses and graphical representations were all carried out in the Minitab® statistics software (version 17; Minitab Inc., State College, USA). The proportion of shoals reorienting at the probe trial was first tested against chance levels (0.5) by a binomial-proportion test. Speed–accuracy trade-offs were tested by rank correlations between time to decide and the number of erroneous trials during training (Spearman's *r_s_*) (Chittka et al., 2009). Decision times from initial and probe trials were found to be normally distributed. Comparisons between trials where any splitting occurred and trials where no splitting occurred was tested by Welch's *t*-test, which does not assume equal variance and sample size. Individuals could not be identified during collective tests because the week-long group acclimation period prevented us from continuously tracking them, and methods of tagging were unavailable. As a result, we could not identify particular individuals with a known boldness score, but we could compare groups of differing composition in terms of individual member boldness. Therefore, regression models (linear for decision times, Poisson for number of days to learn and number of erroneous trials during training, and binary logistic for splitting probability) tested whether each measure was predicted by the mean (5% trimmed to limit bias by minority fish with extreme phenotypes) or the mean absolute deviation of shoal-member boldness (variance across all fish). Individuals with personality tendencies on the extreme ends of our distribution, mostly very shy individuals (Fig. 1), can skew both the mean and variance, making it impossible to assess them as having a different effect, i.e. effects by the slowest individual would appear both in the mean and variance. However, by removing the extreme ends of the group (5% trimmed) we extracted mean values for shoals that represent the majority of their members and not biased by a single very timid fish. Conversely, the variance measure includes these extreme personalities. This enabled differentiation between effects by the majority average (trimmed mean) and the extremes (variance). Models testing decision speed and splitting additionally tested differences between initial and probe trial (categorical predictor; effect of learning) and included shoal number as a random effects term to avoid pseudoreplication. *Post-hoc* comparisons of consistency in splitting were carried out for boldness measures that were found related to splitting, using a one way ANOVA to test if shoals which had split in one, two or zero trials differed in boldness measures.

### Ethical note

All applicable animal-welfare guidelines were followed (ASAB, 2016). Veterinary inspections by DHSSPS, Northern Ireland, deemed no need for licensing. Following the conclusion of the study, animals were kept for separate non-invasive tests.

## References

[BIO033613C1] Bennett A. T. (1996). Do animals have cognitive maps? *J. Exp. Biol.* 199, 219-224. 8576693 10.1242/jeb.199.1.219

[BIO033613C2] Brown C., Laland K. and Krause J. (2006). *Fish cognition and behavior*. Oxford: Blackwell Publishing.

[BIO033613C3] Burgess N. (2006). Spatial memory: how egocentric and allocentric combine. *Trends Cogn. Sci.* 10, 551-557. 10.1016/j.tics.2006.10.005 17071127

[BIO033613C4] Chittka L., Skorupski P. and Raine N. E. (2009). Speed–accuracy tradeoffs in animal decision making. *Trends. Ecol. Evol.* 24, 400-407. 10.1016/j.tree.2009.02.010 19409649

[BIO033613C5] Conradsen C. and McGuigan K. (2015). Sexually dimorphic morphology and swimming performance relationships in wild-type zebrafish Danio rerio. *J. Fish Biol.* 87, 1219-1233. 10.1111/jfb.12784 26416508

[BIO033613C6] Conradt L. and Roper T. J. (2005). Consensus decision making in animals. *Trends Ecol. Evol.* 20, 449-456. 10.1016/j.tree.2005.05.008 16701416

[BIO033613C7] Couzin I. D. (2009). Collective cognition in animal groups. *Trends Cogn. Sci.* 13, 36-43. 10.1016/j.tics.2008.10.002 19058992

[BIO033613C8] Couzin I. D., Krause J., Franks N. R. and Levin S. A. (2005). Effective leadership and decision-making in animal groups on the move. *Nature* 433, 513-516. 10.1038/nature03236 15690039

[BIO033613C9] Croft D. P., Arrowsmith B. J., Bielby J., Skinner K., White E., Couzin I. D., Magurran A. E., Ramnarine I. and Krause J. (2003). Mechanisms underlying shoal composition in the Trinidadian guppy, *Poecilia reticulata*. *Oikos* 100, 429-438. 10.1034/j.1600-0706.2003.12023.x

[BIO033613C10] Croston R., Kozlovsky D. Y., Branch C. L., Parchman T. L., Bridge E. S. and Pravosudov V. V. (2016). Individual variation in spatial memory performance in wild mountain chickadees from different elevations. *Anim. Behav.* 111, 225-234. 10.1016/j.anbehav.2015.10.015

[BIO033613C11] de Perera T. B. and Guilford T. (1999). The social transmission of spatial information in homing pigeons. *Anim. Behav.* 57, 715-719. 10196063 10.1006/anbe.1998.1024

[BIO033613C12] Dyer J. R. G., Croft D. P., Morrell L. J. and Krause J. (2009). Shoal composition determines foraging success in the guppy. *Behav. Eco.* 20, 165-171. 10.1093/beheco/arn129

[BIO033613C13] Griffin A. S., Guillette L. M. and Healy S. D. (2015). Cognition and personality: an analysis of an emerging field. *Trends Ecol. Evol.* 30, 207-214. 10.1016/j.tree.2015.01.012 25736691

[BIO033613C14] Guillette L. M., Naguib M. and Griffin A. S. (2016). Individual differences in cognition and personality. *Behav. Process.* 134, 1-3. 10.1016/j.beproc.2016.12.001 27923604

[BIO033613C15] Hoare D. J., Couzin I. D., Godin J. G. and Krause J. (2004). Context-dependent group size choice in fish. *Anim. Behav.* 67, 155-164. 10.1016/j.anbehav.2003.04.004

[BIO033613C16] Ioannou C. C. and Dall S. R. (2016). Individuals that are consistent in risk-taking benefit during collective foraging. *Sci. Rep.* 6, 33991 10.1038/srep33991 27671145 PMC5037426

[BIO033613C17] Ioannou C. C., Singh M. and Couzin I. D. (2015). Potential leaders trade off goal-oriented and socially oriented behavior in mobile animal groups. *Am. Nat.* 186, 284-293. 10.1086/681988 26655156

[BIO033613C18] Kao A. B., Miller N., Torney C., Hartnett A. and Couzin I. D. (2014). Collective learning and optimal consensus decisions in social animal groups. *PLoS Comput. Biol.* 10, e1003762 10.1371/journal.pcbi.1003762 25101642 PMC4125046

[BIO033613C19] Kareklas K., Elwood R. W. and Holland R. A. (2017). Personality effects on spatial learning: comparisons between visual conditions in a weakly electric fish. *Ethology* 123, 551-559. 10.1111/eth.12629

[BIO033613C20] Krause J., Hoare D., Krause S., Hemelrijk C. K. and Rubenstein D. I. (2000). Leadership in fish shoals. *Fish. Fish.* 1, 82-89. 10.1111/j.1467-2979.2000.tb00001.x

[BIO033613C21] Laland K. N. and Williams K. (1997). Shoaling generates social learning of foraging information in guppies. *Anim. Behav.* 53, 1161-1169. 10.1006/anbe.1996.0318 9236013

[BIO033613C22] Mamuneas D., Spence A. J., Manica A. and King A. J. (2015). Bolder stickleback fish make faster decisions, but they are not less accurate. *Behav. Ecol.* 26, 91-96. 10.1093/beheco/aru160

[BIO033613C23] Marques J. C., Lackner S., Félix R. and Orger M. B. (2018). Structure of the zebrafish locomotor repertoire revealed with unsupervised behavioral clustering. *Curr. Biol.* 28, 181-195.e5 10.1016/j.cub.2017.12.002 29307558

[BIO033613C24] Marras S., Killen S. S., Lindström J., McKenzie D. J., Steffensen J. F. and Domenici P. (2015). Fish swimming in schools save energy regardless of their spatial position. *Behav. Ecol. Sociobiol.* 69, 219-226. 10.1007/s00265-014-1834-4 25620833 PMC4293471

[BIO033613C25] McAroe C. L., Craig C. M. and Holland R. A. (2016). Place versus response learning in fish: a comparison between species. *Anim. Cogn.* 19, 153-161. 10.1007/s10071-015-0922-9 26385107

[BIO033613C26] McAroe C. L., Craig C. M. and Holland R. A. (2017). Shoaling promotes place over response learning but does not facilitate individual learning of that strategy in zebrafish (*Danio rerio*). *BMC Zool*. 2, 10 10.1186/s40850-017-0019-9

[BIO033613C27] McDonald N. D., Rands S. A., Hill F., Elder C. and Ioannou C. C. (2016). Consensus and experience trump leadership, suppressing individual personality during social foraging. *Sci. Adv.* 2, e1600892 10.1126/sciadv.1600892 27652342 PMC5023318

[BIO033613C28] Miller N. Y. and Gerlai R. (2011). Shoaling in zebrafish: what we don't know. *Rev. Neurosci.* 22, 17-25. 10.1515/rns.2011.004 21615258

[BIO033613C29] Miller N., Garnier S., Hartnett A. T. and Couzin I. D. (2013). Both information and social cohesion determine collective decisions in animal groups. *Proc. Nat. Acad. Sci.* 110, 5263-5268. 10.1073/pnas.1217513110 23440218 PMC3612658

[BIO033613C30] Packard M. G. and McGaugh J. L. (1996). Inactivation of hippocampus or caudate nucleus with lidocaine differentially affects expression of place and response learning. *Neurobiol. Learn. Mem.* 65, 65-72. 10.1006/nlme.1996.0007 8673408

[BIO033613C31] Pitcher T. J. and Parrish J. K. (1993). Functions of shoaling behaviour in teleosts. In *The Behaviour of Teleost Fishes* (ed. Pitcher T. J.), pp. 294-337. New York: Chapman & Hall 10.1007/978-94-011-1578-0

[BIO033613C32] Rodriguez F., Duran E., Vargas J. P., Torres B. and Salas C. (1994). Performance of goldfish trained in allocentric and egocentric maze procedures suggests the presence of a cognitive mapping system in fishes. *Learn. Behav.* 22, 409-420. 10.3758/BF03209160

[BIO033613C33] Simons A. M. (2004). Many wrongs: the advantage of group navigation. *Trends Ecol. Evol.* 19, 453-455. 10.1016/j.tree.2004.07.001 16701304

[BIO033613C34] Snekser J. L., Ruhl N., Bauer K. and McRobert S. P. (2010). The influence of sex and phenotype on shoaling decisions in zebrafish. *Int. J. Comp. Psychol.* 23, 70-81.

[BIO033613C35] Sumpter D. J. (2006). The principles of collective animal behaviour. *Philos. T. Roy. Soc. B.* 361, 5-22. 10.1098/rstb.2005.1733 PMC1626537 16553306

[BIO033613C36] Tolman E. C., Ritchie B. F. and Kalish D. (1946). Studies in spatial learning. II. Place learning versus response learning. *J. Exp. Psychol.* 36, 221-229. 10.1037/h0060262 20985357

[BIO033613C37] Toms C. N., Echevarria D. J. and Jouandot D. J. (2010). A methodological review of personality-related studies in fish: focus on the shy-bold axis of behavior. *Int. J. Comp. Psychol.* 23, 1-25.

[BIO033613C38] Trompf L. and Brown C. (2014). Personality affects learning and trade-offs between private and social information in guppies, *Poecilia reticulata*. *Anim. Behav.* 88, 99-106. 10.1016/j.anbehav.2013.11.022

[BIO033613C39] Ward A. J. W., Thomas P., Hart P. J. and Krause J. (2004). Correlates of boldness in three-spined sticklebacks (*Gasterosteus aculeatus*). *Behav. Ecol. Sociobiol.* 55, 561-568. 10.1007/s00265-003-0751-8

[BIO033613C40] Ward A. J. W., Sumpter D. J., Couzin I. D., Hart P. J. and Krause J. (2008). Quorum decision-making facilitates information transfer in fish shoals. *Proc. Natl. Acad. Sci. USA* 105, 6948-6953. 10.1073/pnas.0710344105 18474860 PMC2383955

[BIO033613C41] Ward A. J. W., Herbert-Read J. E., Sumpter D. J. and Krause J. (2011). Fast and accurate decisions through collective vigilance in fish shoals. *Proc. Natl. Acad. Sci. USA* 108, 2312-2315. 10.1073/pnas.1007102108 21262802 PMC3038776

[BIO033613C42] Wolf M. and Weissing F. J. (2012). Animal personalities: consequences for ecology and evolution. *Trends Ecol. Evol.* 27, 452-461. 10.1016/j.tree.2012.05.001 22727728

